# Condom and Substance Use at Last Sex: Differences between MSMO and MSWO High School Youth

**DOI:** 10.3390/ijerph15050995

**Published:** 2018-05-15

**Authors:** Gregory Phillips, Mariah M. Kalmin, Blair Turner, Dylan Felt, Rachel Marro, Paul Salamanca, Lauren B. Beach

**Affiliations:** 1 Department of Medical Social Sciences, Feinberg School of Medicine, Northwestern University, Chicago, IL 60611, USA; blair.turner@northwestern.edu (B.T.); dylan.felt@northwestern.edu (D.F.); rmarro@northwestern.edu (R.M.); paul.salamanca@northwestern.edu (P.S.); lauren.beach@northwestern.edu (L.B.B.); 2 Department of Epidemiology, Gillings School of Public Health, University of North Carolina, Chapel Hill, NC 27599, USA; mariah.kalmin@gmail.com

**Keywords:** HIV, condom use, substance use, alcohol use, YMSM

## Abstract

HIV disproportionately impacts youth, particularly young men who have sex with men (YMSM), a population that includes subgroups of young men who have sex with men only (YMSMO) and young men who have sex with men and women (YMSMW). In 2015, among male youth, 92% of new HIV diagnoses were among YMSM. The reasons why YMSM are disproportionately at risk for HIV acquisition, however, remain incompletely explored. We performed event-level analyses to compare how the frequency of condom use, drug and/or alcohol use at last sex differed among YMSMO and YMSWO (young men who have sex with women only) over a ten-year period from 2005–2015 within the Youth Risk Behavior Survey (YRBS). YMSMO were less likely to use condoms at last sex compared to YMSWO. However, no substance use differences at last sexual encounter were detected. From 2005–2015, reported condom use at last sex significantly declined for both YMSMO and YMSWO, though the decline for YMSMO was more notable. While there were no significant differences in alcohol and substance use at last sex over the same ten-year period for YMSMO, YMSWO experienced a slight but significant decrease in reported alcohol and substance use. These event-level analyses provide evidence that YMSMO, similar to adult MSMO, may engage in riskier sexual behaviors compared to YMSWO, findings which may partially explain the increased burden of HIV in this population. Future work should investigate how different patterns of event-level HIV risk behaviors vary over time among YMSMO, YMSWO, and YMSMW, and are tied to HIV incidence among these groups.

## 1. Introduction

HIV disproportionately impacts youth, particularly young men who have sex with men (YMSM), a group that includes both young men who have sex with men only (YMSMO) and young men who have sex with men and women (YMSMW) [1,2]. In 2015, gay and bisexual young men (aged 13–24 years) made up 92% of new HIV diagnoses among all young men in the same age range [3]. Despite advances in treatment and care outcomes, less is known about what is driving the spread of HIV among YMSM. Therefore, there is a pressing need to understand the nuances of HIV risk behavior interactions among YMSM in order to develop the most effective intervention strategies. One critical area of focus is inconsistent condom use among YMSM, and dynamics that may influence YMSM’s choices about condom use.

Research on disparities in the prevalence of condom use between adult MSMO, MSMW, and men who have sex with women only (MSWO) has yielded mixed results. Although several studies have found that condom use among MSM was higher than among MSW, with estimates ranging from 26–63% for MSM and 12–38% for MSW [4,5,6], lower condom use associated with high-risk sexual behaviors remains elevated in MSM. In a systematic review from 2014, Friedman et al. found that MSMW were significantly less likely than MSMO to engage in condomless receptive anal sex [7]. In 2009, Gorbach et al. found that when specifically comparing anal sex behaviors between the two groups, MSMO were less likely to report using condoms than MSWO [8]. That same study also found that MSMW reported more condomless sex with their female partners than with their male partners, but more condomless anal sex (CAS) with their male partners [8]. Critically, all of these findings point to notable differences in condom usage with male and female partners, particularly as it relates to HIV risk behavior. Data among young men reflect a similar concern—one analysis of 2005–2007 New York City data on high school students showed that YMSMO were less likely than YMSWO to have used a condom during their last sexual encounter (62.4% vs. 79.8%, respectively) [9]. Compared to YMSMO and YMSWO in this study, YMSMW reported the lowest overall condom use; only 44.1% reported using a condom at last sex [9].

One prevailing theory of the driver of lower condom usage with male partners is the relationship of alcohol/substance use with sexual risk behaviors. A number of studies conducted throughout the past several decades have consistently reported associations between alcohol or substance use and increased sexual risk behavior, including decreased condom usage among adult MSM [10,11,12,13,14,15]; this research has been replicated in adolescent populations [16,17,18,19]. Notably, less work has focused on discrepancies among YMSMO, YMSMW, and YMSWO: it is unclear whether lower condom usage in these populations is rooted in the same drivers, or if YMSMO face unique risks for suboptimal condom use compared to YMSWO. Given the disproportionate HIV burden among YMSM, this gap in the literature requires addressing. Based on the evident association between substance use and sexual risk behavior among adult MSM, the influence of alcohol and other substance use on condom use is a logical starting point for exploring differences between YMSMO, YMSMW, and YMSWO. Some studies of sexual risk behavior among MSM have indeed found that, for younger MSM, alcohol use before sex is associated with higher sexual risk [20]. However, the precise nature of this association has not been fully explored.

Recent research has begun to use event-level analysis in order to clarify the relationship between CAS and concurrent substance use. In adult populations, event-level analyses have consistently demonstrated the impact of alcohol and substance use on sexual risk behavior, with same-day alcohol and substance use correlating heavily with decreased condom use, particularly among adult MSM [21,22,23,24]. Unfortunately, similar research into event-level alcohol/substance use and condom use among YMSM remains limited. In 2017, Feinstein and Newcomb reported that same-day drinking among YMSM correlated with CAS with casual partners [25]. To date, no other studies with YMSM have replicated these findings, although same-day alcohol use has been found to be correlated with sexual behavior among YMSM [26]. Further, no studies in YMSM populations have used immediate event-level data of last sex for analysis.

In addition to the demonstrated need for clarity regarding the association between condom and alcohol/substance use among sexually active young males at the event level, data are also lacking on changes in these behaviors over time. Results from the national Youth Risk Behavior Survey (YRBS) indicate that, among sexually active students in grades 9 through 12, prevalence of condom usage at last sex decreased from 2003 to 2015 (63.0 to 56.9%); similar decreases were seen in alcohol and substance use at last sex during that time (25.4 to 20.6%) [27]. However, it is unknown if these trends persist for sexual minority young males as well.

In this paper, we conducted an event-level analysis of alcohol and substance use at the last sexual encounter among sexually active YMSMO and YMSWO. Due to limitations with question wording in the YRBS, we were unable to assess behaviors of YMSMW. We used a large dataset of high school youth (YRBS) with the specific goals of: (1) Comparing the prevalence of condom and substance use at last sex between YMSMO and YMSWO; (2) Exploring the event-level association between these behaviors; and (3) Investigating longitudinal changes in these behaviors between YMSMO and YMSWO over a period of 10 years. Based on the present literature, we hypothesized that: (1) YMSMO will show significantly less condom use and significantly more alcohol/substance use at last sex when compared with YMSWO; (2) Substance and alcohol use will be negatively associated with condom use at the event level of last sex; and (3) Trends over time will show decreasing condom and substance use at last sex among both YMSMO and YMSWO.

## 2. Materials and Methods

### 2.1. Data Source

The YRBS is a biennial national survey that has been conducted by the Centers for Disease Control and Prevention (CDC) since 1991 to collect health data on students in grades 9–12 [28]. The YRBS monitors priority health-related behaviors among youth, such as alcohol use, experiences with violence, suicidal ideation, drug use, sexual behaviors, and eating habits, among others [29]. For this study, we used data from local versions of the YRBS, which are administered on a state, large urban school district, or county level by departments of education or health; in this implementation, jurisdictions use a two-stage cluster sample design to identify a sample of students [28]. In the first stage, schools are selected with a probability proportional to their enrollment; in the second stage, classes of a required subject or during a required period are randomly selected, and all students within these classes are eligible to participate. A new sample is selected in this manner each year that the survey is administered; the same students are not followed over time.

### 2.2. Analytic Sample

Local YRBS data were pooled across multiple jurisdictions (city and state) and years (biennially from 2005–2015). Jurisdictions that asked at least one question about sexual minority status from years 2005–2015 were included. The entire dataset consists of 47 jurisdictions across 10 years, and 541,410 adolescents. The present analysis includes data from the 115 jurisdiction-years that assessed sexual behavior, limited to male students who reported sexual intercourse with males only or females only (85,130 students). Students were excluded if they were missing outcome variables or primary demographic variables of interest (condom use: 15.68%; drug/alcohol use: 15.74%; race/ethnicity: 1.72%; and grade: 2.22%, not mutually exclusive), resulting in a final analytic sample of 63,838 students.

### 2.3. Measures

Sex. Sex was determined by asking participants “*What is your sex?*” with the choices: “*Male*” or “*Female*.” The YRBS does not ask about gender identity; however, we have referred to “male”- or “female”-identified participants as “men” and “women” in this paper for the sake of consistent terminology (see below).

Sex of Partner. Students were asked, “*During your life, with whom have you had sexual contact?*” Students could respond “*I have never had sexual contact*”, “*Females*”, “*Males*” or “*Females and males*”. Males who selected males were considered men who have sex with men only (YMSMO). Male students who selected females were considered men who have sex with women only (YMSWO).

Condom Use. Students were asked, “*The last time you had sexual intercourse, did you or your partner use a condom?*” Response options were, “*I have never had sexual intercourse*”, “*Yes*”, or “*No*”. Students who never had sexual intercourse were set to missing. 

Drug/Alcohol Use. Students were asked, “Did you drink alcohol or use drugs before you had sexual intercourse the last time?” Response options were, “I have never had sexual intercourse”, “Yes”, or “No”. Students who never had sexual intercourse were set to missing.

Race/Ethnicity. Students were asked if they identified as Hispanic or Latino. Additionally, participants could select all races that applied from the list of “American Indian or Alaska Native”, “Asian”, “Black or African American”, “Native Hawaiian or Other Pacific Islander”, and “White”. Categories were collapsed to create the following four race/ethnicity categories: “*White*”, “*Black*”, “*Hispanic/Latino*”, and “*Other Races*”.

Grade. Participants were asked, “*In what grade are you?*” Potential response options were “*9th grade*”, “*10th grade*”, “*11th grade*”, “*12th grade*”, and “*Ungraded or other grade*”. Students who selected “*Ungraded or other grade*” (*n* = 156) were dropped from analysis.

### 2.4. Statistical Analysis

All data cleaning and recoding was conducted in SAS Version 9.4 (SAS Institute, Cary, NC, USA). Analyses were carried out using SAS-Callable SUDAAN Version 11.0.1 (RTI International, Research Triangle Park, NC, USA) to appropriately weight estimates and to account for the complex sampling design of the YRBS. The YRBS data weights adjust for student non-response and distribution of students by grade, sex, and race/ethnicity in each jurisdiction [28]. 

Descriptive statistics were calculated for all variables. Next, logistic regression was used to conduct bivariate analyses between sex of partner and demographic variables. Multivariable logistic models estimated odds of sex of partner associated with condom use and drug/alcohol use at the event-level of student’s last sexual encounter, controlling for race/ethnicity and grade. Then logistic models were run to test for significance of the interaction between condom use and drug/alcohol use at last sex.

Finally, 2005–2015 trends in condom use and drug/alcohol use at last sex, by sex of partner, were assessed with logistic regression. As recommended by the CDC, time was modeled as a continuous variable using orthogonal coefficients to reflect the biennial spacing of the surveys [30,31]. The linear time component was considered significant at *p*-value < 0.05. These analyses controlled for grade and race/ethnicity, and assessed linear, quadratic, and cubic trends. Linear trends test for significant linear increases or decrease over time, while quadratic and cubic trends test for significant non-linear changes over time.

## 3. Results

Within the pooled dataset of 63,838 male high school youth, 3.3% of males reported sex with only other males, whereas 96.7% reported sex with only females (Table 1). The majority of the sample was White (58.71%), with 20.3% identifying as Black and 24.7% identifying as Hispanic/Latino. A slightly larger proportion of individuals were in the 12th grade (33.1%), compared with 9th (17.5%), 10th (22.5%), and 11th grade (26.9%).

### 3.1. Condom Use/Substance Use at Last Sex

Nearly one-third of male youth (30.0%) reported not using a condom at their last sexual encounter. A smaller proportion (21.3%) indicated that they had used drugs and/or alcohol before or during their last sexual encounter. Among all participants, 43.5% reported either not using a condom or using substances at their last sex, with 7.8% reporting both behaviors. 

### 3.2. Associations with Sex of Partner

YMSMO were significantly more likely than YMSWO to identify as Black and significantly more likely to identify as another race/ethnicity, when compared with White identity (odds ratio (OR) = 1.26; 95% confidence interval (CI): 1.01, 1.59; OR = 1.50; 95% CI: 1.13, 1.99, respectively). No grade comparisons were significantly different between the groups (Table 2).

Within a multivariable model controlling for grade and race/ethnicity, we tested the significance of the interaction between condom use and drug/alcohol use at last sex, but found no evidence for effect modification (*p* = 0.58; Table 3). After removing the interaction term, we found that YMSMO were significantly less likely than YMSWO to have used condoms at last sex (adjusted OR (aOR) = 0.39; 95% CI: 0.33, 0.46), but there was no difference for drug/alcohol use at last sex (aOR = 1.06; 95% CI: 0.84, 1.33).

As the YRBS question did not differentiate between alcohol and drug use at last sex, we explored whether the associations could be further disarticulated by looking at lifetime alcohol and drug use. We examined lifetime drug use and lifetime alcohol, disaggregated, as well as lifetime alcohol and drug use combined. Of males who reported using alcohol/drugs before sex, only a small portion had only used alcohol (13.9%) or only used drugs (2.7%) in their lifetime. Due to the increasingly small cell sizes (*n* < 50), we were unable to conduct further sub-analyses.

### 3.3. Longitudinal Associations

**Figure 1:**
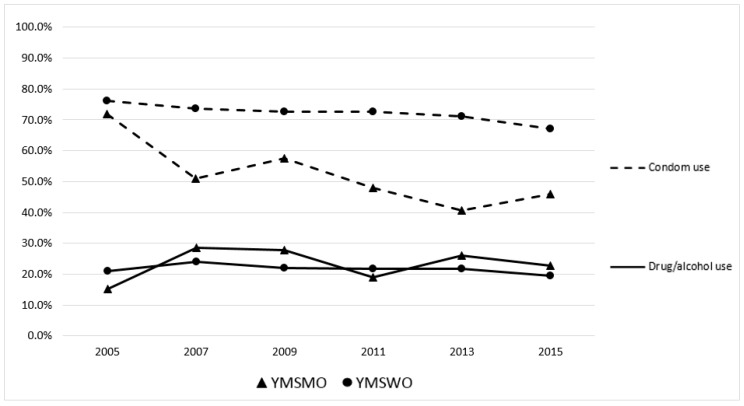
Condom and drug/alcohol use at last sex among YMSMO and YMSWO (YRBS, 2005–2015).

Between 2005 and 2015, the proportion of YMSMO and YMSWO who reported condom use at last sex significantly declined (YMSMO: 71.9 to 46.0%, *p* = 0.001; YMSWO: 76.3 to 67.1%, *p* < 0.001), after controlling for race/ethnicity and grade (Figure 1). Although there was no evidence for significant trends in drug/alcohol use before sex among YMSMO (*p* = 0.81), there was a significant decrease between 2005 and 2015 among YMSWO (21.1 to 19.5%, *p* = 0.004), after controlling for race/ethnicity and grade.

## 4. Discussion

In this analysis of event-level and longitudinal condom-use and substance-use behavior, YMSMO were less likely to use condoms at last sex compared to YMSWO, but no substance use differences at last sexual encounter were found between the two groups. Over a ten-year period, reported condom use at last sex significantly declined for both YMSMO and YMSWO, although the decline for YMSMO was more notable. While there were no significant differences in alcohol and drug use at last sex over the same ten-year period for YMSMO, YMSWO experienced a slight decrease in reported alcohol and drug use that was significant. In this sample, where the overwhelming majority of males reported sex with females, we saw a moderate proportion of participants reporting at least one high risk behavior at last sexual encounter, with only a relatively small proportion of participants reporting both alcohol/drug use and no condom use at last sex.

These results among a geographically diverse sample of sexually active male youth in the US have many notable implications for HIV risk. Although the overall proportion of YMSMO reporting condomless intercourse at last sex (51.8%) is lower than what has been reported among adult MSMO at last sex (69% in a multi-cycle study of data from the NSFG [32]), we did observe a significant decline in reported condom use at last sex over a ten-year period among YMSMO. These event-level analyses provide evidence that YMSMO, similar to adult MSMO, may engage in riskier sexual behaviors compared to YMSWO, findings which may partially explain the increased burden of HIV in this population. Although we hypothesized that no condom use would co-occur with alcohol and drug use, we did not find evidence for this high-risk pairing at last sexual encounter. Therefore, although YMSMO reported a higher prevalence of condomless sex compared to YMSWO, this difference did not seem to be driven by increased alcohol or drug use during the encounter.

Our study had a number of limitations, many of which are associated with inherent challenges in using data collected within YRBS. YRBS does not assess the sex of sexual partner at last sex. Therefore, in order to determine condom use and alcohol/drug behaviors at last sex with male and female partners in our analyses, YMSMW, who comprise an equally large proportion of YMSM as YMSMO within YRBS, were excluded from our analyses. This omission represents a vital gap in our knowledge of event-level HIV risk behaviors among YMSMW and significantly detracted from our ability to examine event-level HIV risk behaviors among YMSM overall. Analyses of lifetime sexual behavior within the 2005–2007 New York City YRBS have shown YMSMW are more likely than YMSMO or YMSWO to engage in drug/alcohol use and are the least likely to use a condom at last sex, suggesting that YMSMW may be the sub-population of YMSM behaviorally at the highest risk for HIV [9]. YMSMW within the New York City YRBS have been shown to be over 4 times more likely to be involved in a teen pregnancy than YMSWO [33]. Few, if any, studies have assessed how HIV risk behaviors may differ among high school aged YMSMW with their male and female partners; it is unknown how the addition of YMSMW into our dataset would have affected the event-level ratios of male/female condom and substance use at last sex. Furthermore, no large, nationally representative analyses have examined the relative prevalence of HIV among YMSMW vs. YMSMO. Taken together, these findings indicate that limited data exist to assess how event-level patterns of condom use, substance use, and sexual behavior affect HIV risk among YMSM overall, though our study does begin to assess these outcomes among the 50% of YMSM who are behaviorally YMSMO.

While assessing condom use and alcohol/drug use at the most recent encounter provides a unique opportunity to investigate event-level associations, it also poses a limitation in understanding the broader sexual history of YRBS participants. For instance, youth could have had other sexual encounters that had different patterns of associated risk behaviors, and these behaviors could have been driven by factors that were not measured within YRBS (e.g., length of relationship, partner age, and, as discussed, partner sex). This is important to note in general, as the relationship between substance use and condomless sex may not be consistent across partner types. One study of young people found that drinking increased the likelihood of condomless sex with casual partners, but not with serious or steady partners [34]. The sex of sex partner may also affect condom use; in addition to HIV prevention, pregnancy prevention may be an important factor affecting decision-making surrounding condom-use behaviors among high-school-aged youth, especially among YMSMW and YSMWO. On a related note, we were restricted to using behavior to define sexual minority status, and were therefore unable to examine how sexual identity might influence both condom and substance use behaviors. In addition, although we could assess trends over time among the populations surveyed, YRBS only contains cross-sectional data; thus, we were unable to assess longitudinal sexual health patterns within the same study participants.

With regard to our specific analyses, we had limited numbers of youth who engaged in both condomless sex and alcohol/drug use at last sexual encounter, resulting in limited power to find an interaction between these two behaviors. Small numbers also prevented us from carrying out sub-analyses to assess whether we could potentially disentangle alcohol and drug use from the compound question used within YRBS. Furthermore, because YRBS is only representative of youth attending schools, our findings may not be generalizable to all members of this age group. For example, previous research has found that youth who do not attend school are more likely to engage in high-risk behavior, and these youth would not be included in a high school-based survey [35]. Lastly, as always with self-reported data, there may be bias, especially under-reporting with regard to risky sexual behaviors, that should be considered.

There were also several strengths associated with our analyses. We had a large sample of male youth over a ten-year period that enabled appropriate comparison of YMSMO and YMSWO in terms of sexual risk behaviors that have been understudied to date. These methods should have been effective at ensuring internal validity [28]. Despite limitations within the YRBS dataset, our findings significantly advance knowledge of event-level patterns of condom use and substance use at last sex among YMSMO vs. YMSWO. This detailed information provides new context for understanding HIV risk behaviors among YMSMO, a population known to be at disproportionately high risk for HIV. 

## 5. Conclusions

Future research should investigate event-level HIV behavioral risk factors among YMSMW. The motivations for condom use and substance use overall and at last sex among YMSMO, YMSMW, and YWSMO should also be investigated, as should type of sexual encounter (oral, anal, and/or vaginal sex). These studies should account for how sex of sexual partner and other partner characteristics may influence YMSM’s decision-making to engage in these and other HIV risk behaviors. Future work should also investigate how different patterns in event-level data for HIV risk behaviors vary over time among YMSMO, YMSMW, and YMSWO and are tied to HIV incidence and prevalence within these groups. Collectively, these findings indicate the need for evidence-informed, culturally tailored interventions, such as sexual minority-inclusive comprehensive sex education, to decrease HIV, sexually transmitted infections (STIs), and pregnancy involvement risk among male sexual minority youth [33,36,37,38]. Improvements in the scope and quality of the sexual minority cultural competency of healthcare providers as well as the development and dissemination of protocols designed to improve HIV prevention among adolescents are also warranted. 

## Figures and Tables

**Table 1:** Characteristics of sexually active males *: 2005–2015 Pooled Youth Risk Behavior Survey (*n* = 63,838).

	*n*	% **
**Sex of Partner**		
Male	2207	3.3
Female	61,631	96.7
**Condom Use at Last Sex**		
Yes	45,339	70.0
No	18,499	30.0
**Drug/Alcohol Use at Last Sex**		
Yes	13,573	21.3
No	50,265	78.7
**Condom & Drug/Alcohol Use at Last Sex**		
Condom use & no drug/alcohol use	36,554	56.5
Condom use & drug/alcohol use	8785	13.5
No condom use & no drug/alcohol use	13,711	22.2
No condom use & drug/alcohol use	4788	7.8
**Grade**		
9th	11,442	17.6
10th	15,044	22.5
11th	18,140	26.9
12th	19,212	33.1
**Race/Ethnicity**		
White	26,548	48.7
Black	13,053	20.3
Hispanic/Latino	16,853	24.7
Other Race	7384	6.3

* Limited to students who reported only male or only female sex partners; ** Weighted frequencies.

**Table 2:** Logistic model estimating the odds of male vs. female sex partner at last sex associated with grade and race/ethnicity: 2005–2015 Pooled Youth Risk Behavior Survey Data. Results significant at *p* < 0.05 are in bold.

	β	SE	*p*-Value	OR	95% CI
**Race/Ethnicity**					
White	REF				
Black	0.24	0.11	**0.046**	**1.26**	**(1.00, 1.59)**
Hispanic	0.16	0.16	0.309	1.18	(0.86, 1.61)
Other	0.41	0.14	**0.005**	**1.50**	**(1.13, 1.99)**
**Grade**					
9th	-0.18	0.14	0.209	0.84	(0.63, 1.10)
10th	-0.25	0.13	0.056	0.78	(0.60, 1.01)
11th	0.04	0.13	0.735	1.05	(0.81, 1.35)
12th	REF				

**Table 3:** Multivariable logistic regression model estimating odds of male vs. female partner at last sex: 2005–2015 Pooled Youth Risk Behavior Survey Data. Results significant at *p* < 0.05 are in bold.

	M1: No Interaction					M2: Interaction Model				
	β	SE	*p*-Value	OR	95% CI	β	SE	*p*-Value	OR	95% CI
**Condom Use at Last Sex**										
Yes	−0.95	0.08	<0.001	**0.39**	**(0.33, 0.46)**	−0.97	0.09	<0.001	**0.38**	**(0.32, 0.45)**
No	REF									
**Drug/Alcohol Use at Last Sex**										
Yes	0.05	0.12	0.650	1.06	(0.84, 1.33)	0.00	0.15	0.980	1.00	(0.75, 1.35)
No	REF									
**Grade**										
9th	−0.15	0.15	0.318	0.86	(0.65, 1.15)	−0.15	0.15	0.323	0.86	(0.65, 1.15)
10th	−0.19	0.14	0.162	0.83	(0.63, 1.08)	−0.19	0.14	0.164	0.83	(0.63, 1.08)
11th	0.08	0.14	0.558	1.08	(0.83, 1.41)	0.08	0.14	0.556	1.08	(0.83, 1.41)
12th	REF									
**Race/Ethnicity**										
White	REF									
Black	0.33	0.12	0.007	**1.39**	**(1.10, 1.77)**	0.33	0.12	0.007	**1.39**	**(1.10, 1.77)**
Hispanic	0.16	0.17	0.352	1.17	(0.84, 1.64)	0.16	0.17	0.352	1.17	(0.84, 1.64)
Other	0.42	0.15	0.004	**1.53**	**(1.14, 2.05)**	0.43	0.15	0.004	**1.53**	**(1.14, 2.05)**
**Condom Use × Drug/Alcohol Use**						0.11	0.2	0.580	1.12	(0.75, 1.66)
